# Caregiver Burden among Parents of Hearing Impaired and Intellectually Disabled Children in Pakistan

**Published:** 2020-02

**Authors:** Imran Haider SYED, Waqar Ahmed AWAN, Unaiza Batool SYEDA

**Affiliations:** 1. Department of Sociology, Allama Iqbal Open University, Islamabad, Pakistan; 2. Riphah College of Rehabilitation & Allied Health Sciences, Riphah International University, Islamabad, Pakistan; 3. Helping Hand Institute of Rehabilitation Sciences, Mansehra, Pakistan

**Keywords:** Disability, Deaf, Hearing impairment, Intellectually challenged

## Abstract

**Background:**

Caregiver burden is a multidimensional response to physical, psychological, emotional, social and financial stressors, usually associated with the experience of caring and can be objective or subjective. The objective of current study was **t**o explore the caregiver burden among parents of hearing impaired and intellectually challenged children in Pakistan.

**Methods:**

A Comparative cross sectional survey was conducted on n=162 parents of hearing impaired (HI) and intellectually challenged (IC) children from July 2018 to February 2019. Convenient sampling technique was used to collect the data from Parents of hearing impaired and intellectually challenged children with age range 1–16 years in National Institute of Rehabilitation Medicine and Al-Farabi Special Education Institute Islamabad. Caregiver Burden Inventory was used to assess the caregiver burden.

**Results:**

The results showed a greater need for respite and other services in both groups. Parents of intellectually challenged children need more respite and other services as compared to hearing impaired children (60.62±11.43 ver. 45.74±11.20, p<0.001). A total of 3 (4.0%) parents of hearing impaired children reported rare need for respite and other services, 32(42.7%) reported sometimes and 40(53.3%) reported frequent need. On the other hand 12(13.8%) parents of intellectually disabled children reported sometimes, 66(75.9%) reported quite frequently and 9(10.3%) nearly always a greater need for respite and other services.

**Conclusion:**

The parents of hearing impaired or intellectually challenged children face significant burden of their disabled child. In addition, due to cognitive deficits that lead to behavioural abnormalities the parents of intellectually challenged children face more burden and stress.

## Introduction

The yoke of caregiver is a multifaceted approach to psycho-sociological strains that are generally linked with the patterns of caring and are looked in with respect to objectivity as well as subjectivity. Measurable effects such as economic issues, caregivers’ loss of work or social and leisure activities, household disruptions, restrictions on relationships within and outside the family etc. are included in objective burden while the psychological sufferings in the form of depression, hatred, uncertainty, guilt, shame, and embarrassment all are included in subjective burden (1, 2). Parents having children with hearing impairment and intellectually disabled tend to be disturbed with added responsibilities associated with care of their children in the normal day to day functions (3).Hearing impairment is a common abnormality by birth, and is more than twice as prevalent as others which are screened for in new-borns worldwide. The impairment may inflict the newborn during or shortly after birth due to genetic factors, trauma or disease. In most advanced countries, 2–4 out of every 1000 births are inflicted with moderate to severe hearing loss congenitally or postnatally. Severe to profound hearing loss prevails among more than 10 out of every 1000 babies. The hearing loss pathology is of multiple natures as it can be either one-sided or two-sided with similar level of hearing loss severity in both ears that is referred to as symmetrical hearing loss or there might be variation in the severity of hearing loss that is known as asymmetrical hearing loss. The diagnosis of hearing loss is a critical life event with profound effects on parents and the family system with deep stress (4).

Initial diagnosis of hearing impairment brings difficulties for most of the parents, other family members and hence the entire family system in the form of mental stress due to financial and time strains. Communicating with such children gets difficult especially when condition is accompanied by delayed speech, intellectual disability and communication development, which may lead to social stigma and isolation that entails them with feelings of grief, disappointment, helplessness, aggression. In addition to coping with the shock of the initial diagnosis, families must acquire a comprehensive understanding of how to manage the affected child, through getting information on hearing aids, sign language, educational methods, school placements, and legal issues (5–7).

Almost 156 million people worldwide have been afflicted with intellectual disability which is said to be a huge problem throughout the world because of its complex medical, psychological, social, educational and legal aspects, therefore is considered as a difficult problem to define, understand, educate, and manage to everybody’s fulfilment at various levels of disability in the society (8). Intellectual disability is a disorder with an onset during the developmental period that includes both intellectual and functioning deficits in the cognitive, social and practical domains. According to DSM 5, an approximately 1% of the overall general population suffers from intellectual disability. Approximately 0.12% of 3- to 5-year-old children and 0.62% of 6- to 21-year-old children suffer from intellectual disability (2, 9).The intellectual disability may entail emotional, economic and social disruption and restriction on parents with most noticeable problems such as denial in accepting intellectually challenged children, care giving fatigue, issues in maintaining relationships, financial problems, feeling embarrassment by the child’s violent behaviour in public places (6, 10, 11).

Some features such as the nature of disabilities, behavioural problems of children, emotional status, lack of social support, and stressful resources associated with the inability of children may cause increasing stress among parents (12). Psychological abnormalities of this type make care-giving burdensome. These children often need special care because of delaying or failure to obtain customs such as toilet training, dressing, eating, clothing, and problems such as bed-wetting and seizures. In order to achieve diagnosis and treatment, parents become hasty and strict, and this might result in financial loss, and a loss of energy and time. They cannot take proper care of themselves or other members of the family (13). Constant efforts of care giving may create perception of pressure among the care givers that lead to psychological pathologies like shame, remorse, excessive load, feeling oneself to be caged, bitterness, isolationism and lack of control internally as well as externally. (14–16).

Hence, the aim of this study focuses to explore the care giver burden among parents of children with hearing impairment and intellectually disability in Pakistan.

## Methods

A Cross sectional survey was conducted to explore caregiver burden on parents of hearing impaired and intellectually challenged children. Overall, 162parents of children with hearing impairment and intellectual disability were included. Non-probability convenient sampling technique was used to collect data.The data was collected from Al-Farabi Special Education Institute and National Institute of Rehabilitation Medicine, Islamabad after approval from Principal and Executive Director, respectively. The duration of the study was 7 month from July 2018–February 2019. Parents of hearing impaired and intellectually challenged children with age range of 1–16 years were included while parents of children with any other systemic co-morbidity or disability other than hearing impairment and intellectual disability were excluded. The data was collected through demographic forms including age of child & caregiver, gender of child & caregiver, caregiver education, occupation & income, family system and severity of disability.

Care giver burden was evaluated through Care-giver Burden Inventory. Multidimensional measures of caregiver burden gave a sensitive reading of caregivers’ feelings and a sophisticated picture of caregivers’ responses to the demands of care on 24-item, five-subscale Caregiver Burden Inventory (CBI) (17).

The results of study have been presented as frequency, percentages and mean ±SD. To determine the difference between HI & ICC regarding caregiver burden independent t-test was performed. The data was analysed through SPSS V 21 (Chicago, IL, USA).

## Results

**Fig. 1:**
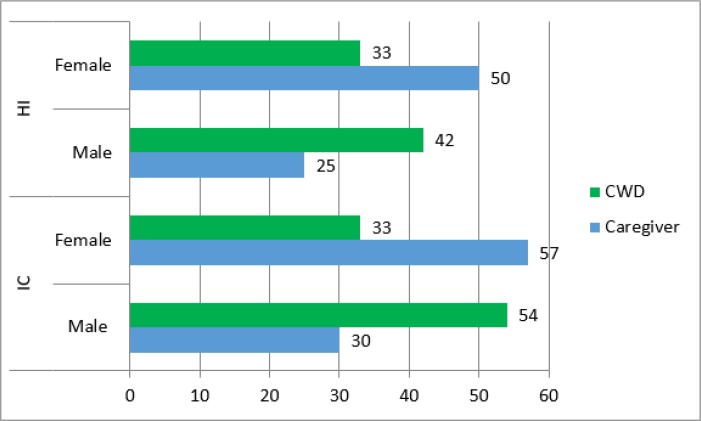
Gender of CWD & Caregivers

Minimum age of child with disability was between 1 to 16 years. The mean age of children with disability (CWD) was 9.26±4.309. A total n=87 children were intellectually challenged(IC) and n=75 were hearing impaired (HI). The mean age of the HI children and IC children was8.36±4.25 and 10.30±4.16 respectively. Age range of parents of disabled children was between 25 to 60 years with41.50±6.41 (mean ±SD).The mean age of the parents of HI children and IC children was41.83±6.79 and 41.12±5.96 respectively. The detail frequency of gender regarding caregivers and CWDs can be seen in Fig. 1.

**Fig. 2:**
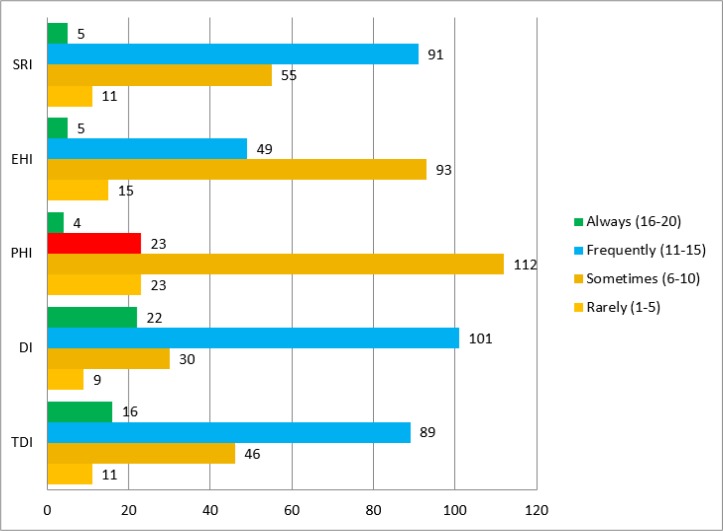
Caregiver burden (Sub domains) ***TDI****=Time Dependency Items,* ***DI****= Development Items,* ***PHI****= Physical Health Items,* ***EHI****= Emotional Health Items,* ***SRI****=Social Relationship Items*

The mean score of time dependency item (12.43±3.67), development item (13.23±3.30) and social relationship items (10.57± 2.88) showed that majority of caregiver frequently need respite care. While mean score of physical health (8.13±2.71) and emotional items (9.30±3.21) showed that majority of caregiver sometime need respite care to manage their physical (n=93) and emotional burden (n=112). The difference between mean score of HI and IC children regarding sub domain of caregiver burden inventory can be seen in Table 1 (Fig. 2).

**Table 1:** Difference between IC (n=87) & HI (n=75) children regarding subdomain of CBI

***Variable***	***Disability***	***N***	***Mean***	***SD***
TDI	IC	87	14.05	2.92
	HI	75	10.56	3.58
DI	IC	87	14.11	2.95
	HI	75	12.21	3.42
PHI	IC	87	9.43	2.80
	HI	75	6.62	1.60
EHI	IC	87	11.21	2.62
	HI	75	7.08	2.27
SRI	IC	87	11.70	2.28
	HI	75	9.26	2.96
Total CBI	IC	87	60.62	11.43
	HI	75	45.74	11.20

***TDI****=Time Dependency Items,* ***DI****= Development Items,* ***PHI****= Physical Health Items,* ***EHI****= Emotional Health Items,* ***SRI****= Social Relationship Items.*

P<0.0001

**Fig. 3:**
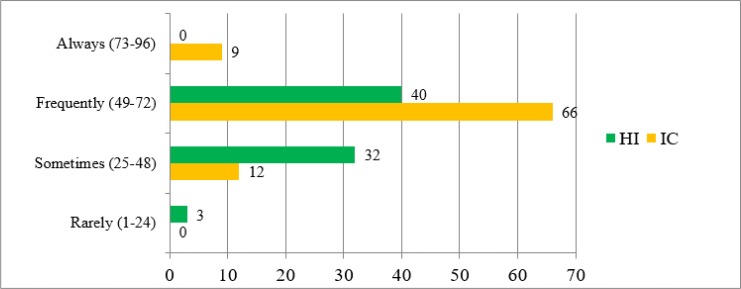
Caregiver burden (Frequency distribution)

The result showed a greater need for respite and other services in both group. The overall caregiver burden inventory score was 53.73±13.52, which showed n=106(65.43%) participant frequently need respite and other services to manage caregiver burden. Parents of intellectually challenged children need more respite and other services as compare to hearing impaired children (60.62±11.43 ver. 45.74±11.20, *P*<0.001) (Table 1). The frequency distribution of caregiver burden in both groups can be seen in Fig. 3.

## Discussion

The present study aimed to explore the caregiver burden among the parents of hearing impaired (HI) and intellectually challenged (IC) children. The study showed high score on caregiver burden inventory which indicate the caregiver of both HI and IC children frequently need respite care for coping their burden. It also includes significantly different levels of caregiver burden among two different groups of HI and IC children.

Parents of children with HI exhibited significantly moderate level of stress. Results are consistent with a study in the USA on the impact of duration of hearing loss on parental stress. The PHICE was administered to 152 caregivers of children with permanent hearing loss and the result showed higher stress levels (18).In another study, mothers of the deaf or hard of hearing reported greater life stress as compared to those of normal hearing infants (19). Besides, higher level of stress was experienced by the care giver parents of deaf and dumb children in comparison to others (20). Another study indicated that mothers of the hearing and orthopedically impaired have high level of hopelessness and depression (21)

Parents of children with ID showed severe to profound levels of stress. A study from India showed higher level of daily parenting stress and anxiety among parents of children with intellectual disability (22). In Taiwan, mothers with school-aged intellectually disabled children had a rather high level of strain (23). In Nairobi, parents of children with ID were likely to be at risk for depression and to be highly stigmatized, tend to have higher-than-average rates of stress, anxiety and depression and an increased risk of family dysfunction, and a number of physical and mental health conditions due to chronic psychological stress (24)*.*

Another result of our study showed that parents of children with HI and ICC exhibited significantly high levels of caregiver burden.An study showed an increase in total care giving associated with a younger child who was more physically and mentally impaired, requiring more medical treatment, resulting in higher level of caregiver burden (25). Literature on caregiver’s challenges and quality of care towards mentally retarded children showed that Caregivers’ emotional, social and financial constraints can adversely affect their quality of care (1, 26). Carpinello et al showed considerable subjective and objective burden among parents of mentally retarded children and of neurologically impaired children, while no difference was seen in the level of burden (27).

One of the findings from current study was that parents of children with ID had severe to profound levels of psychological stress and caregiver burden as compared to parents of HI (23). Additionally, the results of the study are similar to preceding research studies conducted in India that concluded that care giver parents of children suffering from ADHD and associated developmental pathologies experience more psychological pressure in comparison to care givers parents of children with diseases like asthma and HIV aids. This current study found out that younger and middle hood aged parents experienced more stress which is in consistent with a study that documented younger parents having higher levels of depressive symptoms and older parents having lower level of depression due to experienced parenting and adaptation to stress as the time passes (28).

One of the most striking results of this study was the significant relationship between gender of parents and caregiver burden (*P*=0.05). Results showed higher scores of stress in mothers with (mean score=76) and also felt severe level of caregiver burden with (mean score=64) than fathers.Literature from US and UK showed mothers to be more stress in parent domain than fathers and fathers were less affected by the child’s characteristics than were mothers (29, 30). In Pakistan, mothers had greater proportion of anxiety or depression than fathers (31).

The marital status of the caregiver significantly influences their level burden and stress. The single parents have significant higher level of stress and felt higher level of burden than the parents living in nuclear or joint family system (32, 33). The divorced / separated parents had significant higher levels of depression than the married ones (34) while parents with low level of educational status were highly stressed and felt severe level of caregiver burden as compared to parents with high level of educational status associated with better employment status (business or self-employment), better source of income leads to lower risks of stress and caregiver burden symptoms (29, 35). Another study showed significant relation between depression of the mothers and education level and financial status of the families. Mothers with lower income and education levels were suspected of depression (36).

Parents who had child with profound level of disability were highly stressed and had high level of burden than those parents who had child with other ranges of severity levels (37).More severe disability in the child was associated with greater levels of parenting stress in mothers (30).A direct relationship between severity of childhood disability and parenting stress levels i-e: higher the severity higher the parenting stresses is reported (38).

The current study lags behind in sample size and finding relationship of caregiver burden and factors related to caregiver e.g. age, gender, socioeconomic status, family system etc. that may contribute in caregiver burden. It was also a single centred study so generalizability of the result not justified as Pakistan is diverse in term of socio-economic status, availability of health care services and education level of caregivers’ in different cities of Pakistan.

## Conclusion

Parents of children with disability either hearing impaired or intellectually challenged children face significant burden of their disabled child. It was also concluded that parents of intellectually challenged children face more burden as compare to parents of children with hearing impairment due to cognitive deficits. It is recommended that analytical study should be done to find out relationship among caregiver burden, multiple disabilities of children and gender based differences of caregivers along with quality of life.

## Ethical considerations

Ethical issues (Including plagiarism, informed consent, misconduct, data fabrication and/or falsification, double publication and/or submission, redundancy, etc.) have been completely observed by the authors.
